# Malignant Transformation of Human Bronchial Epithelial Cells Induced by Arsenic through STAT3/miR-301a/SMAD4 Loop

**DOI:** 10.1038/s41598-018-31516-0

**Published:** 2018-09-05

**Authors:** Mingtian Zhong, Zhujuan Huang, Lei Wang, Zhanwen Lin, Zhi Cao, Xun Li, Fengxue Zhang, Hongqi Wang, Yong Li, Xiaodong Ma

**Affiliations:** 1 The Research Center of Basic Integrative Medicine, Guangzhou University of Chinese Medicine, Guangzhou, 510006 China; 2 Institute for Brain Research and Rehabilitation, Guangdong Key Laboratory of Mental Health and Cognitive Science, Center for Studies of Psychological Application, South China Normal University, Guangzhou, 510631 China; 3 Department of Cancer Biology, Lerner Research Institute, Cleveland Clinic, Cleveland, OH USA

## Abstract

Arsenic is a well-known of human carcinogen and miR-301a is an oncogenic microRNA, which links to oncogenesis, however, little is understood about its contribution to arsenic-induced cellular transformation and tumorigenesis. Here, we investigated the role of miR-301a during arsenic-induced cellular transformation and tumor formation. miR-301a was found to be upregulated during arsenic-induced BEAS-2B transformation and the overexpression of miR-301a was dependent on IL-6/STAT3 signaling. Inhibition of miR-301a leads to reduction of cell proliferation, colony formation and cell migration. By using dual luciferase assay, SMAD4 was confirmed to be a direct target of miR-301a in BEAS-2B cells and upregulation of SMAD4 is involved the restraining cell growth and migration. In addition, reducing of miR-301a expression enhances doxorubicin-induced cellular apoptosis of transformed BEAS-2B through up-regulating SMAD4. Furthermore, we demonstrated that downregulation of miR-301a in BEAS-2B attenuates tumor growth in the xenograft model by targeting SMAD4. Of note, the level of miR-301a expression correlated inversely with SMAD4 expression in clinical specimens of human lung cancer. Our findings ascertain that miR-301a is an oncogenic miRNA, which targets SMAD4 to establish an essential mechanism for arsenic-induced carcinogenesis, IL-6/STAT3/miR-301a/SMAD4 signaling pathways.

## Introduction

Arsenic is an established environmental toxicant that exists naturally in drinking water^1^, soil, and food across the world. Chronic exposure to inorganic arsenic has been associated with numerous adverse health outcomes, including lung, skin, kidney, liver, prostate and urinary bladder cancers, skin lesions and cardiovascular disease^2^. Arsenic can induce immortalized human cell line such as BEAS-2B to become malignant transformed cells, which possess the intrinsic properties of cancer cells such as loss of contact inhibition, gain of anchorage-independent growth, resistant to apoptosis, enhance of cellular migration and invasion, and the ability of tumor formation on xenograft mouse model^3^. Several genotoxic and epigenetic alterations have been tightly associated with the arsenic transformation process, which leads to increased cancer risk. Recent advances in the understanding to the fundamental biology of arsenic-induced cellular transformation have led to the epigenetic mechanisms including DNA methylation, Histone modification and aberrant expression of microRNAs.

MicroRNAs (miRNAs), small, non-coding, single-stranded RNA molecules of 19–25 nucleotides, are important controllers of gene expression and regulators of malignant transformation and metastasis^4^. Several miRNAs have been identified in arsenic-induced cellular transformation and carcinogenesis. microRNA array study revealed altered microRNA expression likely controls Ras oncogene activation during malignant transformation of human prostate epithelial and stem cells by arsenic^5^. MiR-200b suppresses arsenic-transformed cell migration by targeting protein kinase Cα (PKCα) and Wnt5b^6^. Knockdown of miR-21 inhibited arsenic-induced human bronchial epithelial cell proliferation and carcinogenesis by targeting PDCD4^7^. Moreover, exposure to arsenic rapidly induces a multifaceted dedifferentiation program and miR-205 has potential to be used as a marker of arsenic exposure as well as a maker of early urothelial carcinoma detection^8^. Over 1000 human miRNAs have been identified so far, miR-301a is a potential oncogenic miRNA and contributes to tumor formation. From the study of cancer cell lines and deficient mouse models of miR-301a indicated that miR-301a regulated cellular malignancy process in multiple cancer including human lung cancer, liver cancer, gastric cancer, pancreatic cancer, colorectal cancer, breast cancer, prostate cancer, glioblastomas, and Laryngeal neoplasms^9–14^. In lung cancer, knockdown of miR-301a reduces anchorage independent colony formation of lung cancer cells and inhibit cellular proliferation, migration and invasion of non-small cell lung cancer cell line^15,16^. However, the biological functions of miR-301a involved in the process of arsenic-induced cellular transformation remain largely uninvestigated.

Our previous studies demonstrated that over-expression of miR-301a contributes to two deadly malignancies: lung cancer and colorectal cancer^10^. Deletion of miR-301a reduced lung tumor development and increases survival in *Kras*^*LA2*^ mice, which correlates with reduced the activation of both NF-κB and STAT3. Interestingly, sustained overproduction of IL-6/STAT3 was found to be contributed to arsenic-induced cellular transformation and carcinogenesis^7,17^. Unlike STAT3, arsenic related upregulation of NF-κB is closely correlated with increased immune-suppression instead of IL-6 upregulation response related cellular transformation^18^. Thus, the mechanisms by which miR-301a modulating STAT3 signaling in the development of arsenic-induced cellular transformation are needed to clarify. In the present study, we reported that miR-301a is over-expressed during the transformation of BEAS-2B cells induced by chronic exposure to arsenic. Further study demonstrated that STAT3/miR-301a/SMAD4 cascade promote the arsenic-induced cellular transformation and tumorigenesis. Silencing of miR-301a or induction of Smad4 in arsenic transformed BEAS-2B cells reduce the tumorigenesis in xenograft nude mice. Thus, our findings suggest that the activation of STAT3/miR-301a/SMAD4 loop is a key positive regulator in human lung bronchial epithelial cells induced by this heavy metal ion arsenic.

## Results

### Arsenic induced the upregulation of miR-301a in BEAS-2B cells

**Figure 1:**
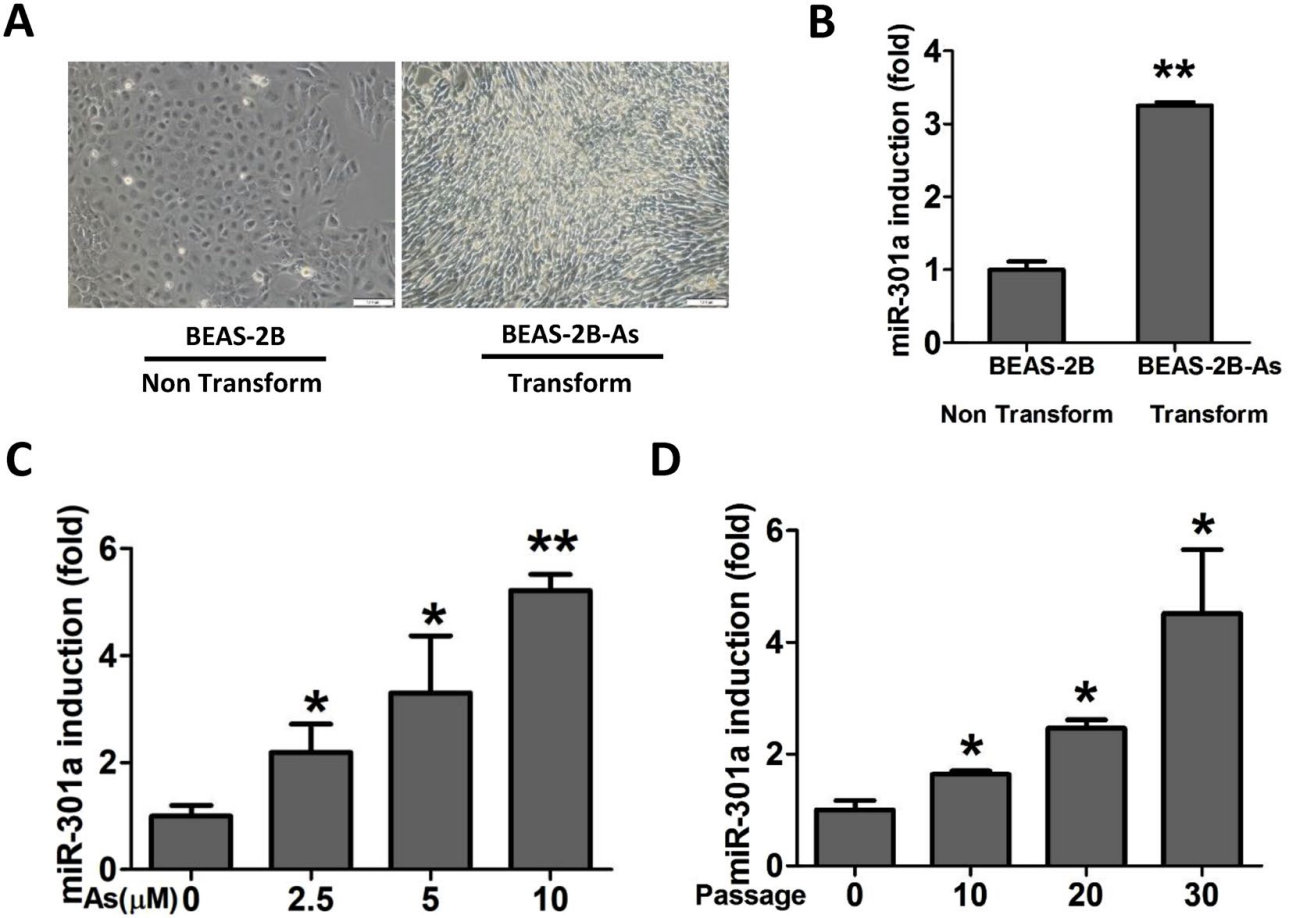
Arsenic induces miR-301a expression during cellular transformation. **(A)** BEAS-2B cells were exposed to arsenic (0.25 μM) for 6 months and the morphology of normal BEAS-2B (BEAS-2B) and transformed BEAS-2B (BEAS-2B-As) cells are present. **(B)** The expression of miR-301a was determined by qPCR between none transformed BEAS-2B (BEAS-2B) and transformed BEAS-2B (BEAS-2B-As) cells. **(C)** BEAS-2B cells were exposed to different concentrations of arsenic (0, 2.5, 5, 10 μM) for 12 h and the expression of miR-301a was determined by qPCR. **(D)** BEAS-2B cells were exposed to 0.25 μM arsenite for 0, 10, 20, 30 passages and the expression of miR-301a was determined by qPCR. Values represented the mean ± SD of three independent experiments. *P < 0.05 and **P < 0.01 indicate a significant difference between none transformed and transformed cells, or between none treatment and treatment groups.

To explore the role of miR-301a during arsenic-induced cellular transformation, we established the transformed BEAS-2B cells. BEAS-2B cells were exposed to arsenic (0.25 μM) up to 6 months, and then the cells were undergoing malignant transformation (Fig. 1A). We firstly measured the expression level of miR-301a between non-transformed BEAS-2B cells and arsenic-induced transformed BEAS-2B cells. miR-301a was highly expressed in transformed BEAS-2B cells compared with non-transformed cells (Fig. 1B). Meanwhile, the expression level of miR-301a in BEAS-2B cells when exposed to different concentrations of arsenic (0, 2.5, 5, or 10 μM) for 12 h were compared. Our results showed that with the increase of the concentrations of arsenic, the expression level of miR-301a was significantly increased, confirming that miR-301a expression was stimulated by arsenic (Fig. 1C). We then compared miR-301a expression in BEAS-2B cells exposed to 1 μM arsenic for 0, 10, 20, 30 passages. With increased time of exposure to arsenic (10–30 passages), and there was greater expression of miR-301a (Fig. 1D). These results indicated that there was increased miR-301a expression with longer times for transformation of BEAS-2B cells and arsenic can induce up-regulation of miR-301a during cellular transformation.

### Arsenic induced miR-301a expression dependent on IL-6/STAT3 signaling

**Figure 2:**
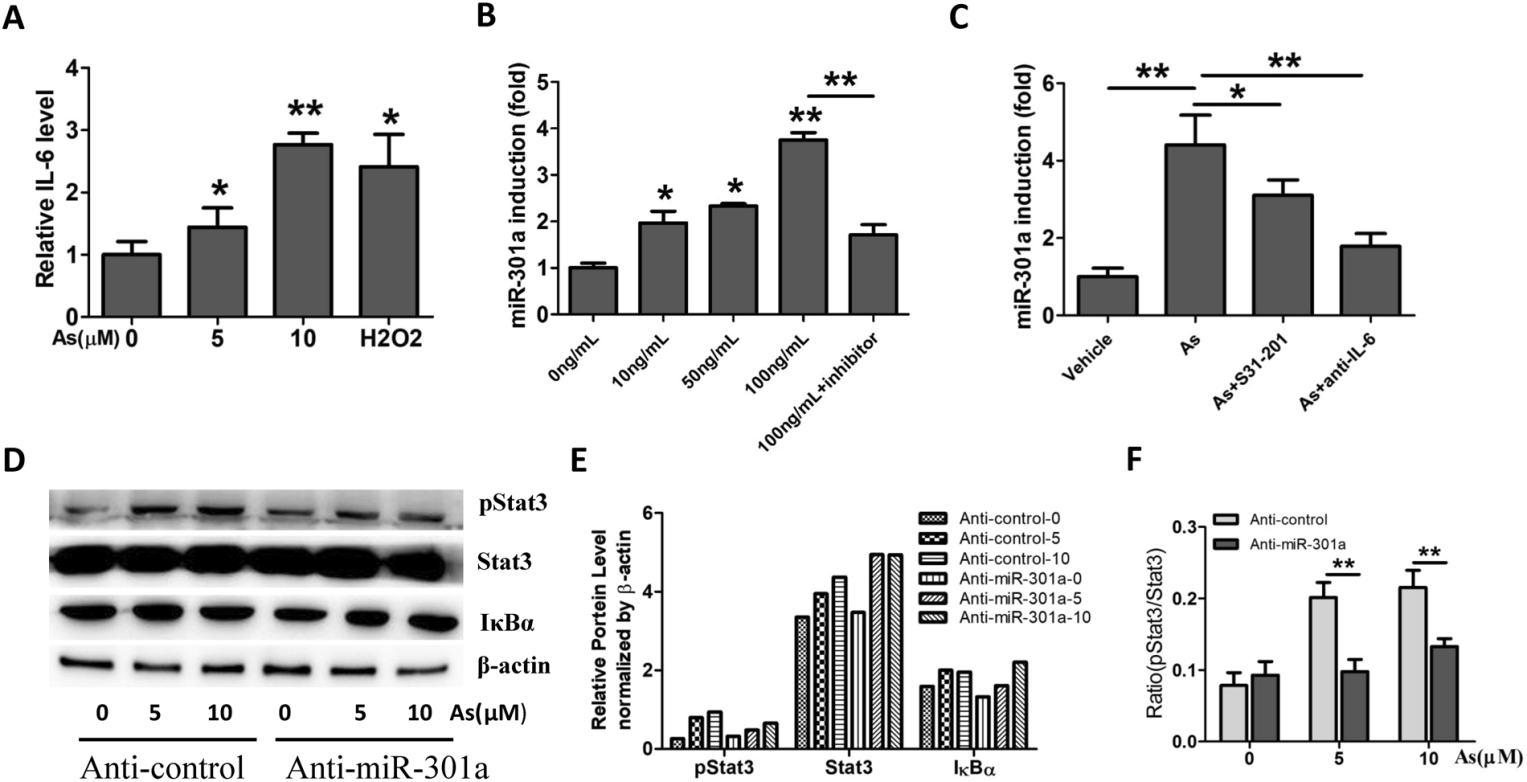
Arsenic induced miR-301a expression dependent on IL-6/STAT3 signaling. **(A)** BEAS-2B cells were treated with arsenic (5 and 10 μM) for 24 h and with H2O2 as positive control. The levels of IL-6 in the culture medium was determined by using ELISA Kit. **(B)** BEAS-2B cells were treated with different concentrations of IL-6 (0, 10, 50, and 100 ng/mL) and IL-6 plus Stat3 inhibitor (S31-201, 30 μM), at 6 h and the expression of miR-301a was determined by qPCR. **(C)** BEAS-2B cells were treated with arsenic (As, 10 μM), arsenic (As) and S31-201, arsenic (As) and IL-6 neutralizing antibody (anti-IL-6) at 6 h and the expression of miR-301a was determined by qPCR. **(D)** BEAS-2B cells were transfected with Anti-control (Anti-control) and LNA-Anti-miR-301a(Anti-miR-301a). After 48 h post transfection, cells were treated with arsenic (5 μM and 10 μM) for 24 h and western blot analyses of phospho-Stat3 (pStat3), Stat3, IκBα expression. Gel pictures were acquired by using Tanon5200 and the exposure time was 60 sec for pStat3, 20 sec for Stat3, 20 sec for IκBα, and 5 sec for β-actin. **(E)** Protein expression from **(D)** were quantified by band intensity and normalized to β-actin. (**F**) pStat3 expression was quantified by band intensity and normalized to total Stat3. Values represented the mean ± SD of three independent experiments. *P < 0.05 and **P < 0.01 indicate a significant difference between the indicated groups, or between none treatment and treatment groups.

IL-6 and STAT3 were recently to be described as pro-tumorigenic mediators in the regulation and maintenance of the cellular transformation^7,19^. In the present study, we measured the levels of IL-6 in the culture medium in BEAS-2B cells exposed to 0, 5, 10 μM arsenic and H2O2 as positive control for 24 h, and found that the level of IL-6 was upregulated at highest level with 10 μM arsenic treatment (Fig. 2A). STAT3 is a downstream target of IL-6, then we determined whether IL-6 mediates miR-301a levels via inducing STAT3 activation in BEAS-2B cells. We stimulated BEAS-2B cells with different concentrations of IL-6 (0, 10, 50, and 100 ng/mL), and measured miR-301a expression. miR-301a was significantly increased with IL-6 stimulation, and the effect was abrogated with STAT3 inhibitor, S31-201 treatment (Fig. 2B). Next, we determined whether arsenic induced the induction of miR-301a was dependent on IL-6 and STAT3. BEAS-2B cells were treated with arsenic in the presence of STAT3 inhibitor and IL-6 neutralizing antibody (anti-IL-6). As we expected, arsenic alone treatment results the higher level of miR-301a, while with inhibition of either STAT3 inhibitor or IL-6 inhibition reduced the expression of miR-301a (Fig. 2C).

As miR-301a was reported to be an activator of both STAT3 and NF-κB in lung tumorigenesis^10^, we sought to determine whether inhibition of miR-301a leads to reduce the activation of Stat3 or NF-κB. We measured the expression of phosphor-STAT3, total STAT3 and IκBα in BEAS-2B cells treated with 5 and 10 μM arsenic for 24 h. We found that arsenic activated STAT3 in BEAS-2B cells, and such activation was markedly inhibited by reduction of miR-301a (Fig. 2D–F). Furthermore, there was no significant differences of IκBα expression between BEAS-2B cells transfected with anti-control and with anti-miR-301a with arsenic treatment (Fig. 2D,E). These data suggest that miR-301a-mediated STAT3 activation is induced by arsenic in BEAS-2B cells, which further confirm that IL-6 mediation of miR-301a levels is related to STAT3 in BEAS-2B cells treated with arsenic.

### Knockdown of miR-301a inhibited cell proliferation, colony formation, cell migration and apoptosis in transformed BEAS-2B cells

**Figure 3:**
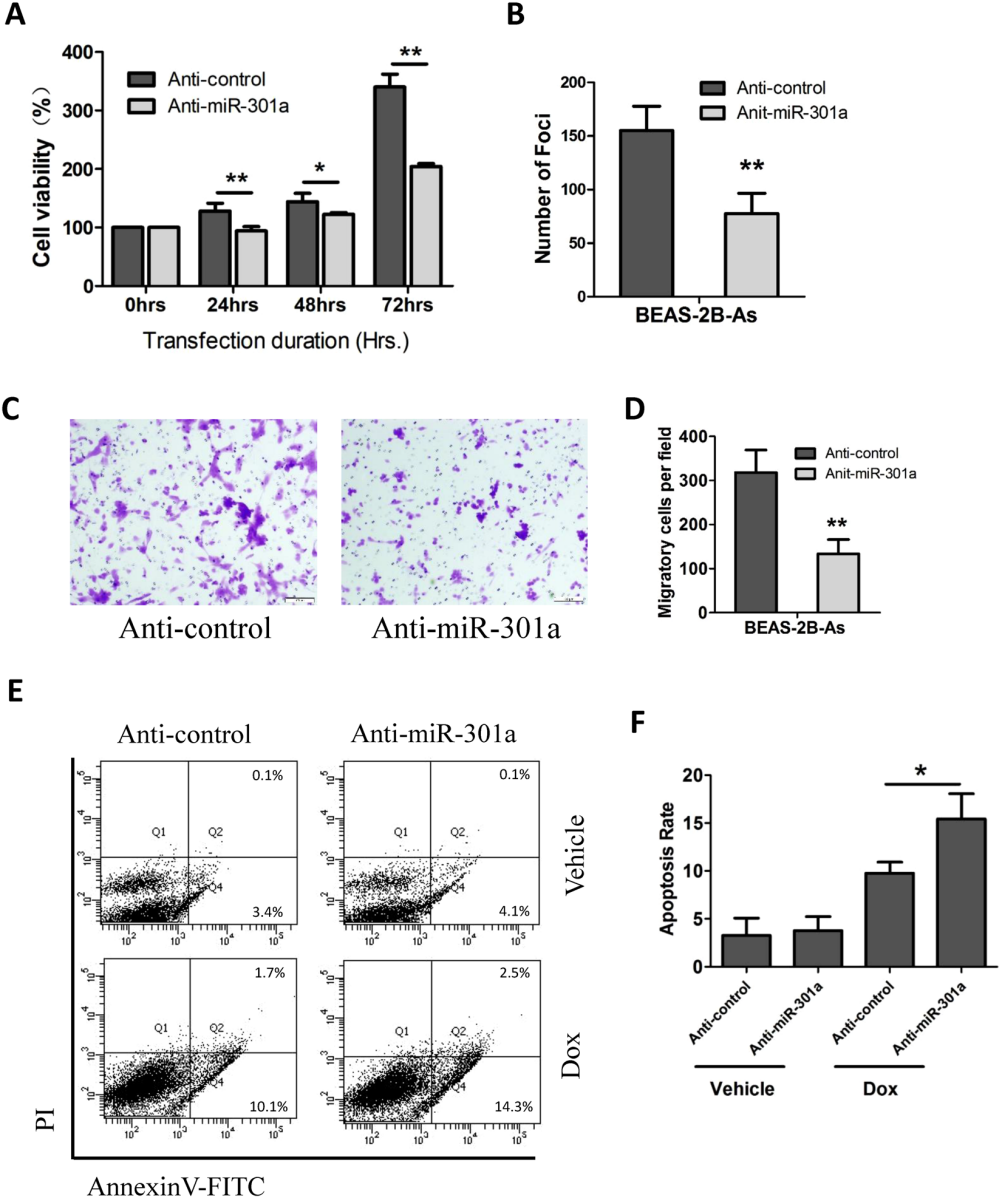
Knockdown of miR-301a inhibited cell proliferation, colony formation, cellular migration and promoted doxorubicin-induced cell apoptosis in arsenic transformed BEAS-2B cell. Arsenic transformed BEAS-2B cells were transfected with Anti-control (Anit-control) or LNA-Anti-miR-301a (Anti-miR-301a). **(A)** Cell proliferation was determined by using cell counting kit-8 at the indicated times. **(B)** Cells were seeded in soft agar for 4 weeks and the number of colonies in the field were counted. **(C)** Cell migration was determined by using trans well assay. (**D**) Migratory cells were counted from **(C)**. **(E)** Cell apoptosis was measured by using annexinv and PI staining. Doxorubicin (0.2ug/ml) was used to induced cellular apoptosis for 24 h. (**F**) Apoptotic cells were counted from **(E)**. Values represented the mean ± SD of three independent experiments. *P < 0.05 and **P < 0.01 indicate a significant difference between Anti-control and Anti-miR-301a transfection groups.

As an oncogenic miRNA, miR-301a was involved in the regulation of various processes associated with a variety of human malignancies, including cell proliferation, viability, metastasis and invasion. To investigate the function of miR-301a in arsenic-induced transformed BEAS-2B cells, we measured the cell proliferation, colony formation, cell migration and apoptosis in transformed BEAS-2B cells with inhibition of miR-301a. Down-regulation of miR-301a, by transfection with the miR-301a inhibitor, significantly restrained the cell viability (Fig. 3A) and reduced the ability of colony formation (Fig. 3B). We then examined whether miR-301a contribute to the migration of malignant transformed cells by performing trans well migration assay. We found that miR-301a inhibitor compromised the migratory ability of transformed BEAS-2B cells (Fig. 3C,D). These results demonstrate that miR-301a reduction inhibits the malignancies of transformed BEAS-2B cells.

miR-301a has been implicated in drug resistance of various tumors, and overexpression of miR-301a reduced doxorubicin-induced cellular apoptosis in osteosarcoma and malignant melanoma^20^. We then determined the cellular apoptosis in transformed BEAS-2B cells responding to DNA damage when altering miR-301a expression. There was no significant difference of the frequency of apoptotic cells between transformed BEAS-2B cells introduced with Anti-control and transformed BEAS-2B cells with anti-miR-301a (Fig. 3E,F). However, inhibition of miR-301a significantly enhanced the chemo-sensitivity of doxorubicin in transformed BEAS-2B. These data confirmed that miR-301a promote cell proliferation and migration of arsenic induced transformed BEAS-2B cells and inhibition of miR-301a would be beneficial in apoptosis-inducing cancer therapies against arsenic-induced tumorigenesis.

### miR-301a Targeting SMAD4 in Transformed BEAS-2B cells

**Figure 4:**
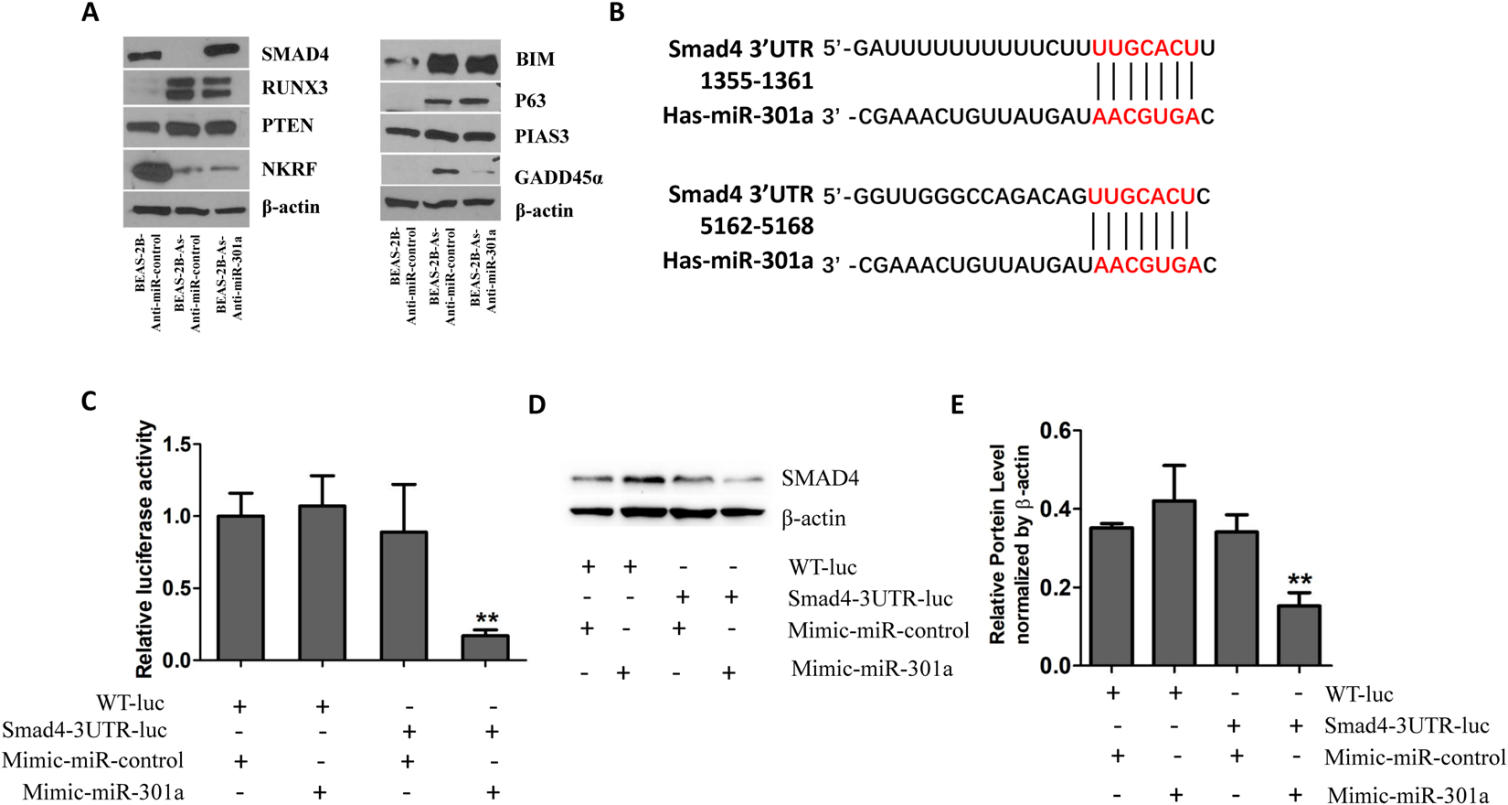
SMAD4 is a direct target of miR-301a in arsenic transformed BEAS-2B cells. **(A)** Western blot analyses of miR-301a targets among none transformed BEAS-2B (BEAS-2B) with anti-miR-control, transformed BEAS-2B (BEAS-2B-As) with Anti-miR-control, and transformed BEAS-2B (BEAS-2B-As) with Anti-miR-301a. **(B)** Prediction of major interference sites between miR-301a and SMAD4 mRNA 3′UTR by using Targetscan. **(C)** Luciferase activity in BEAS-2B-As cells transfected with the indicated luciferase reporter with either an miR-301a mimic or control oligonucleotide. **(D)** Western blot analysis of SMAD4 expression in BEAS-2B-As cells with the indicated luciferase reporter with either an miR-301a mimic or control oligonucleotide. Gel pictures were acquired by using Tanon5200 and the exposure time was 30 sec for SMAD4 and 5 sec for β-actin. Values represented the mean ± S.D. *P < 0.05 and **P < 0.01 indicate a significant difference between the indicated groups.

To identify the potential direct target genes of miR-301a in transformed BEAS-2B cells, we firstly evaluated the protein levels of several reported miR-301a target genes (SMAD4, RUNX3, PTEN, NKRF, BIM, P63, and PIAS3) and one potential target, GADD45α, which is identified as miR-301a target by us (unpublished data). Only SMAD4 and NKRF expression was dramatically downregulated in arsenic-induced transformed BEAS-2B cells than in non-transformed BEAS-2B cells (Fig. 4A). Of note, inhibition of miR-301a expression significantly enhanced the expression of SMAD4 in transformed BEAS-2B cells and did not affect the expression level of NKRF. These data suggest that SMAD4 play a key role during arsenic-induced BEAS-2B cell transformation.

To test the hypothesis that miR-301a directly suppress SMAD4 expression in transformed BEAS-2B cells, we analyzed the binding site for miR-301a and Smad4 RNA 3′UTR. The miRNA target prediction software Targetscan (version 5.2) search revealed the presence of two major binding sites for miR-301a within the Smad4 RNA 3′UTR (Fig. 4B). By using a luciferase reporter system, we sought to determine whether miR-301a directly binds the mRNA encoding Smad4 and down-regulates expression of Smad4. Arsenic-induced transformed BEAS-2B cells were co-transfected with full length Smad4 3′UTR sequence and the miR-301a mimic, the luciferase activity was inhibited by 80% (Fig. 4C) and the protein level of SMAD4 was correspondingly reduced (Fig. 4D,E). Neither transfection of miR-301a mimic control with Smad4 3′UTR nor transfection of miR-301a mimic with WT luciferase into transformed BEAS-2B cells affected the luciferase activity and SMAD4 protein levels (Fig. 4C–E). These data indicated that miR-301a directly targets the mRNA encoding Smad4 and knockdown of miR-301a leads to elevating SMAD4 protein level in arsenic-induced transformed BEAS-2B cells.

### Knockdown of SMAD4 enhance the malignant characteristics of transformed BEAS-2B cells with miR-301a inhibition

**Figure 5:**
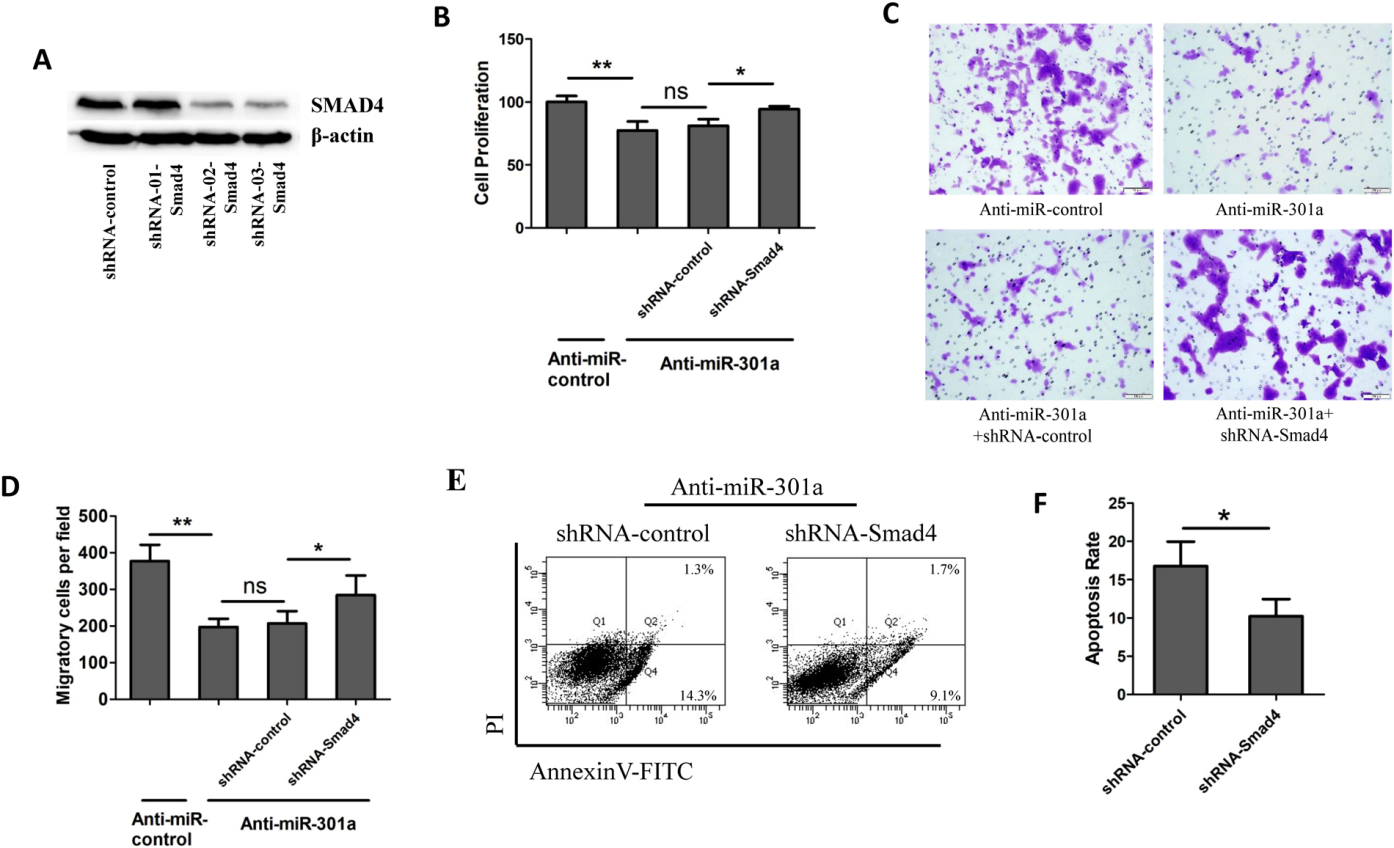
Knockdown of SMAD4 enhance the malignant characteristics of transformed BEAS-2B cells with miR-301a inhibition. **(A)** Western blot shows the efficacy of shRNA knockdown of SMAD4 in BEAS-2B transformed cells. Gel pictures were acquired by using Tanon5200 and the exposure time was 30 sec for SMAD4 and 5 sec for β-actin. **(B)** Cell viability of transformed BEAS-2B cells transfected with Anti-miR-control, or Anti-miR-301a with blank, shRNA control (shRNA-control) and shRNA-Smad4 were determined by cell counting kit-8. **(C)** Transformed BEAS-2B cells were transfected with the indicated inhibitor and cell migration were determined by using trans well assay. **(D)** Migratory cells were counted from **(C)**. **(E)** Cell apoptosis was measured by using annexinv and PI staining. Transformed BEAS-2B cells were transfected with the indicated inhibitor and treated with doxorubicin (0.2 μg/ml) for 24 h. **(F)** Apoptotic cells were counted from **(E)**. Values represented the mean ± SD of three independent experiments. *P < 0.05 and **P < 0.01 indicate a significant difference between the indicated groups. ns represent no significant differences.

Next, we determined whether SMAD4 affect the phenotype of transformed BEAS-2B cells with miR-301a inhibition. We first designed three shRNA against Smad4 and found shRNA-02 and shRNA-03 significantly downregulated SMAD4 expression in transformed BEAS-2B cells (Fig. 5A). Thus, we used the shRNA-03-Smad4 for the following experiments. We then evaluated the cell proliferation by using CCK-8 kit and found that knockdown of Smad4 via short hairpin RNA (shRNA) significantly enhanced cell proliferation (Fig. 5B). Similarly, Smad4 shRNA promotes the ability of migratory of transformed BEAS-2B cells with miR-301a inhibition (Fig. 5C,D). In addition, in transformed BEAS-2B cells transfected with miR-301a inhibitor, Smad4 shRNA reduced the cellular apoptotic death (Fig. 5E,F). Collectively, our results indicated that upregulation of SMAD4 is responsible for the reduced cell proliferation and migration, and enhanced doxorubicin-induced cellular apoptosis observed in transformed BEAS-2B cells with miR-301a inhibition.

### Inhibition of miR-301a reduces tumor growth *in vivo*

**Figure 6:**
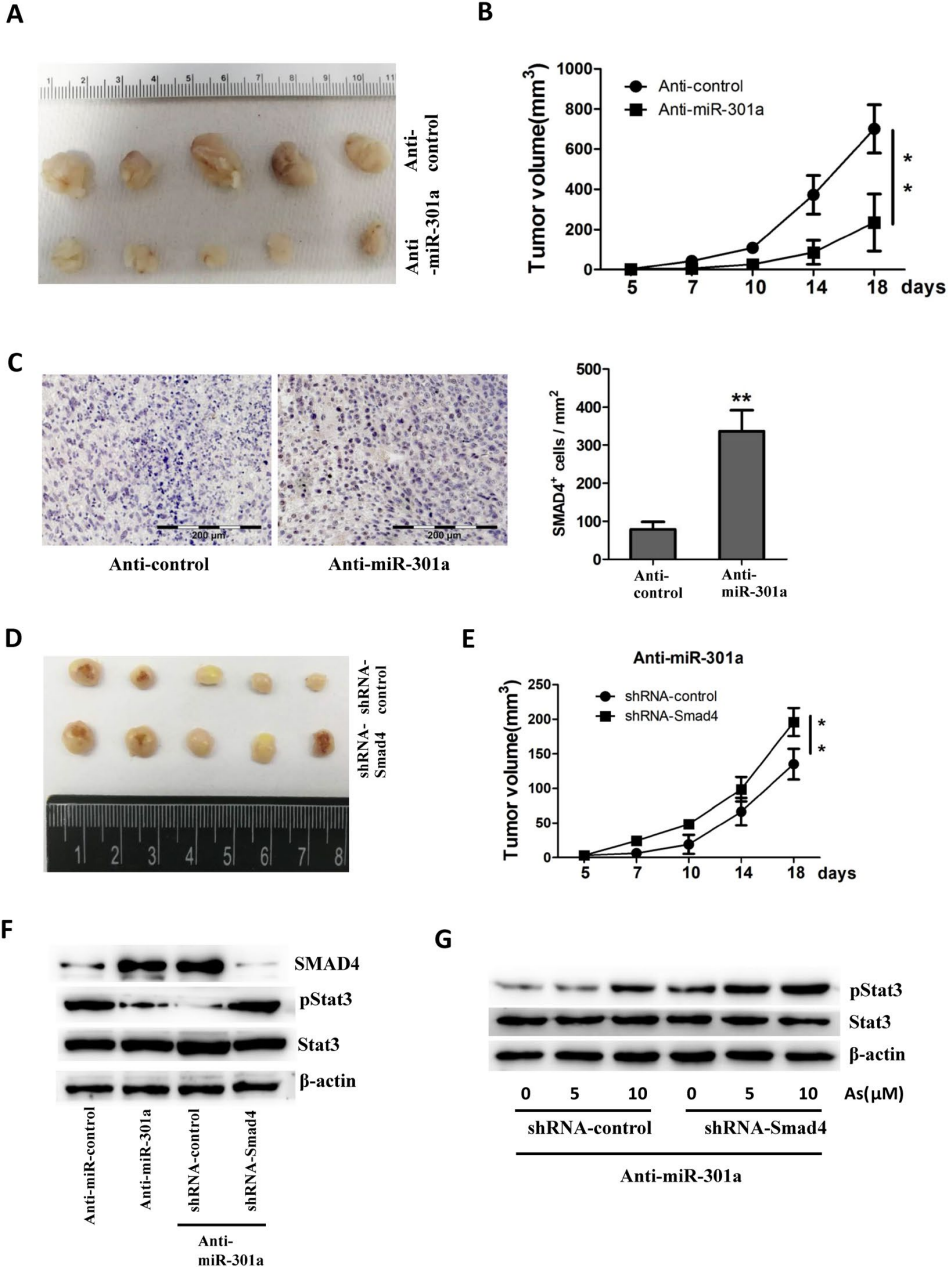
Inhibition of miR-301a reduces tumor growth in mouse xenografts through SMAD4. **(A)** 2 × 10^5^ transformed BEAS-2B (BEAS-2B-As) with Anti-miR-control or Anti-miR-301a were injected subcutaneously into nude mice. Representative images of tumor from nude mice are shown. **(B)** Tumor volume of each group was measured respectively. **(C)** Detecting the expression of SMAD4 in tumors of each group. Scale bars = 200 μm. **(D)** 2 × 10^5^ transformed BEAS-2B (BEAS-2B-As) with Anti-miR-301a plus either shRNA control or shRNA-Smad4 were injected subcutaneously into nude mice. Representative images of tumor from nude mice are shown. (**E**) Tumor volume of each group from **(D)** was measured respectively. **(F)** Western blot analyses of SMAD4, phospho-Stat3 (pStat3) and total Stat3 of tumors from the indicated groups. Gel pictures were acquired by using Tanon5200 and the exposure time was 30 sec for SMAD4, 60 sec for pStat3, 30 sec for Stat3 and 10 sec for β-actin. **(G)** BEAS-2B cells were transfect with the indicated shRNA control or shRNA-Smad4 in the presence with LNA-anti-miR-301a(Anti-miR-301a). After 48 h post transfection, cells were treated with arsenic (5 and 10 μM) for 24 h and western blot analyses of phospho-Stat3 (pStat3) and Stat3 expression. Gel pictures were acquired by using Tanon5200 and the exposure time was 60 sec for p-Stat3, 30 sec for Stat3 and 10 sec for β-actin. Values represented the mean ± SD (n = 5 mice per group). *P < 0.05 and **P < 0.01 indicate a significant difference between the indicated groups.

Given the pro-tumorigenesis exerted by miR-301a during arsenic induced BEAS-2B transformation, we sought to determine the role of miR-301a in tumor growth by using xenograft tumor model. We subcutaneously injected transformed BEAS-2B cells either with Anti-miR-control or Anti-miR-301a into nude mice respectively. Compared to transformed BEAS-2B cells with Anti-miR-control, tumor growth was significantly reduced in transformed BEAS-2B cells with miR-301a inhibition (Fig. 6A,B). Correspondingly, the expression of SMAD4 was distinctly upregulated in the tumor with miR-301a inhibition (Fig. 6C). To define the impact of SMAD4 on tumor growth with miR-301a inhibition, transformed BEAS-2B cells with anti-miR-301a plus either shRNA control or shRNA-Smad4 were subcutaneously injected into nude mice. As we expect, the effect of miR-301a inhibition on restraining tumor growth was reversed by the presence of Smad4 shRNA in transformed BEAS-2B cells (Fig. 6D,E). In addition, we measured the expression of SMAD4, phosphor-STAT3(pStat3), STAT3 in tumors separated from above four groups. Inhibition of miR-301a leads to highly expressed SMAD4 and dramatically attenuated the activation of STAT3, whereas knockdown of SMAD4 was found to restore the activation of STAT3 (Fig. 6F). In consistent with this, knockdown of SMAD4 leads to increase the activation of STAT3 in BEAS-2B cells with miR-301a inhibition treated with arsenic (Fig. 6G). These data support the conclusion that attenuated tumor growth in xenograft model by miR-301a inhibition was due to elevating SMAD4 expression and consequently abating the activation of STAT3.

### miR-301a oppositely correlate with SMAD4 expression

**Figure 7:**
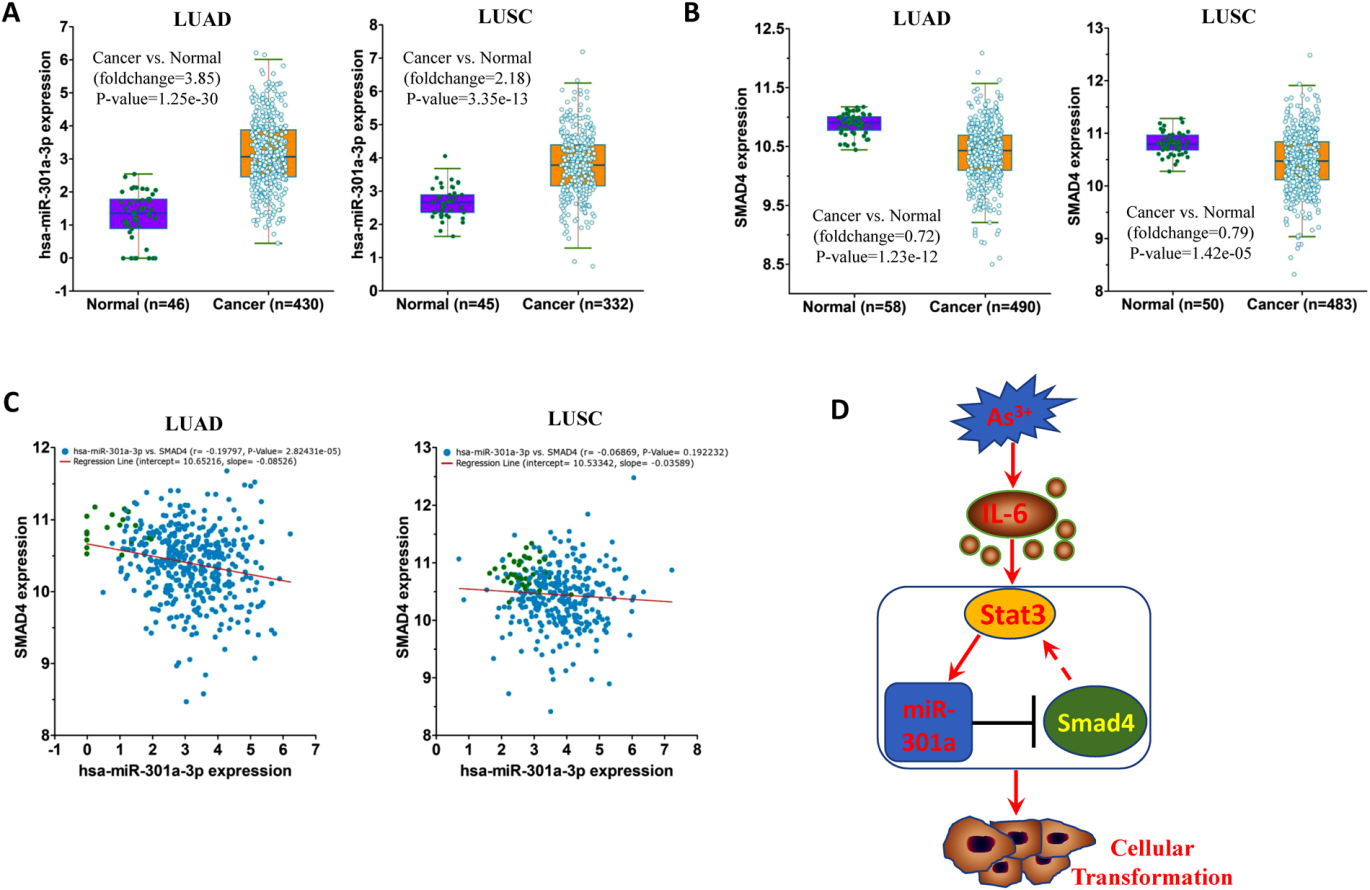
miR-301a and Smad4 expression in human lung adenocarcinoma and squamous cell carcinoma and the correlation analysis. **(A)** miR-301a and **(B)** SMAD4 expression in human lung adenocarcinoma (LUAD) and squamous cell carcinoma (LUSC) samples compared to normal lung samples from TCGA database. **(C)** Correlation analyses between miR-301a and SMAD4 in human lung adenocarcinoma and squamous cell carcinoma samples from TCGA database. **(D)** Schematic of the effects of miR-301a on arsenic induced cellular transformation.

To identify the correlations between miR-301a and SMAD4 in lung cancer of clinical patients from the TCGA (Cancer Genome Atlas) databases, we choose two type of lung cancer, adenocarcinoma (n = 430) and squamous cell carcinoma (n = 332) with normal lung tissue samples to analyze the expression of miR-301a and SMAD4. miR-301a displayed significantly higher expression in both lung adenocarcinoma and lung squamous cell carcinoma compared to normal tissue (Fig. 7A), whereas its target genes, SMAD4 was significantly downregulated in lung cancer sample compared to normal tissue (Fig. 7B). Interestingly, miR-301a also show inverse correlation with SMAD4 in lung adenocarcinoma, while it did not display significant anti-correlation to Smad4 in lung squamous cell carcinoma (Fig. 7C), suggesting the complex network of miRNA regulation during tumorigenesis. These data indicate that miR-301a inversely correlates with Smad4 expression in lung adenocarcinoma and the positive feedback loop between miR-301a and SMAD4 contribute to the cellular transformation and tumorigenesis induced by arsenic (Fig. 7D).

## Discussion

Arsenic is one of the most well-known human carcinogens, and long-term exposure can significantly increase the risk of developing multiple type of cancers including lung, skin, kidney, liver and bladder cancer^3,21^. It is generally accepted that epigenetic mechanism may play a key role in arenic-induced tumorigenesis mostly due to its weakly genotoxic carcinogen^22,23^. Recent findings revealed that noncoding RNAs, especially of microRNAs, have participated in arsenic-induced cellular transformation and tumorigenesis. For example, knockdown of miR-21 reduces the cell proliferation and xenograft tumors produced by arsenic-induced transformed human embryo lung fibroblast cell (HELFs)^24^ and human bronchial epithelial cells (BEAS-2B)^19^. miR-200 family members were reported to play an important role in arsenic caused human cancer by their capability to inhibit epithelial-to-mesenchymal transition (EMT)^8^ and cancer metastasis^6^. In the present work, we investigated the role of miR-301a in arsenic-induced BEAS-2B cellular transformation and tumorigenesis. The major finding of this study is that inhibition of miR-301a reduced the malignance of transformed human bronchial epithelial cells and the activation of STAT3/miR-301a/SMAD4 contribute to the tumorigenesis of arsenic-induced transformed cells.

We showed that miR-301a is not just overexpressed in transformed BEAS-2B cells compared to non-transformed BEAS-2B cells but that it is overexpressed in non-transformed BEAS-2B cells treated with arsenic with time and dose dependent manner. miR-301a was found to be persistently upregulated in different passage of BEAS-2B cells exposed to arsenic, which suggest it’s regulated by some transcript factors whose activation leads to cellular transformation. The promoter for miR-301a contains a κB site and the expression of miR-301a was reported to be regulated by NF-κB in pancreatic cancer cells^25^. Thus, we measured whether NF-κB was activated in BEAS-2B cells treated with arsenic. However, the expression of IκBα, an inhibitor of NF-κB, is not altered by arsenic treatment, suggesting that activated miR-301a is controlled by other transcript factors. Sustained overproduction of IL-6 and STAT3 was found to contributes to arsenic causes carcinogenesis^26,27^ and STAT3 is also required for cellular transformation and performed pro-tumorigenic functions, including promotion of tumor cell proliferation, survival, invasion, metastasis, and angiogenesis^28–30^. Interestingly, miR-301a was shown to be an activator of STAT3 in T cells and lung tumor mouse model through downregulating its target gene, Pias3^10,31^. Here, we have found that, induction by arsenic, secretion of IL-6, a pro-inflammatory cytokine, led to the activation of STAT3, a downstream target of IL-6, and to increased levels of miR-301a. These results suggest a positive feedback loop: miR-301a down-regulates its target genes to elevate STAT3 activation, which in turn promotes miR-301a transcription.

We next investigated which miR-301a target is responsible for the cellular transformation and tumorigenesis caused by arsenic. We found that SMAD4 expression was total lost when BEAS-2B was transformed by arsenic compared to non-transformed cells. In transformed BEAS-2B cells, SMAD4 expression was completely restored by introducing miR-301a inhibitor. Smad4 was found to be a miR-301a target in pancreatic cancer^32^, prostate cancer^33^ and laryngeal squamous cell carcinoma^11^. We for the first time demonstrated that Smad4 is a direct target of miR-301a in arsenic-induced transformed BEAS-2B cells and knockdown of SMAD4 enhanced the malignance of transformed BEAS-2B cells with miR-301a inhibition. Of note, SMAD4 suppresses the phosphorylation of Stat3 in pancreatic cancer cells^34^, which leads us to explorer the connection between SMAD4 and STAT3 in transformed BEAS-2B cells. We reasoned that elevated SMAD4 is responsible for the reducing cellular transformation and tumorigenesis with miR-301a inhibition. We further demonstrated that arsenic treatment elevates the activation of STAT3 in BEAS-2B cells, yet miR-301a inhibition significantly reduces it. Knockdown of SMAD4 by shRNA increases the activation of STAT3 in both transformed cells and xenograft mouse model with the presences of miR-301a inhibitor. These findings suggest that miR-301a modulates arsenic-induced cellular transformation and tumorigenesis through negative regulation of Smad4, and further supports a key role for miR-301a in metal carcinogen-induced carcinogenesis.

Given pulmonary tissue is considered to be the most sensitive site for arsenic toxicity, and long-term arsenic exposure significantly increases the risk of lung cancer^35,36^, we compared the expression level of miR-301a and Smad4 in patients with lung cancer from TCGA. We found miR-301a was upregulated in both lung adenocarcinoma and squamous cell carcinoma, whereas Smad4 show the downregulation in these two types of lung cancer. We only observe a significant inverse correlation between miR-301a and Smad4 levels in lung adenocarcinoma, but not in lung squamous cell carcinoma, which indicate that other potential miR-301a targets probably participant the process of cell transformation and tumorigenesis.

In summary, we present a novel IL-6/STAT3 dependent mechanism for sustained expression of miR-301a during arsenic-induced cellular transformation, and further describe the important role of miR-301a and its target gene, Smad4 in arsenic-induced tumorigenesis. These observations provide a positive feedback loop: STAT3/miR-301a/SMAD4/STAT3 responsible for miRNA participated in the regulation of arsenic-induced carcinogenesis. miR-301a and SMAD4 could be a therapeutic target for preventing and curing the lung cancer developing with chronic exposure of arsenic.

## Methods

### Cell culture and reagents

The human bronchial epithelial cell line BEAS-2B was obtained and authenticated from the Shanghai Institute of Cell Biology, Chinese Academy of Sciences (Shanghai, China). Arsenic transformed cells were generated as described previously^37^. Briefly, BEAS-2B cells were exposed to 0.25 μM As2O3 for 72 h per passage and cultured for 40 passages. BEAS-2B and Arsenic transformed cells were cultured in Dulbecco’s Modified Eagle’s Medium (DMEM) supplemented with 10% fetal bovine serum (FBS), 2mM L-glutamine, and 5% penicillin/streptomycin at 37 °C in a humidified atmosphere with 5% CO_2_ in air. Sodium arsenic (CAS no 7784-46-5, purity: 99%), STAT3 inhibitor, S31-201 and doxorubicin were purchased from Sigma Chemical Corp (Sigma, St. Louis, MO). Human recombinant IL-6 was purchased from R&D Systems (R&D, Minneapolis, MN).

### Quantitative real-time PCR

Total cellular RNA was isolated by using Trizol (Invitrogen) according to the manufacturer’s recommendations. RNA was reverse transcribed with the iScript Reverse Transcription Supermix kit (Bio-Rad, Hercules, CA, USA) following the manufacturer’s protocol. Real-time PCR for miR-301a detection was performed using TaqMan assay (Thermo Fisher) with U6 small nuclear RNA as reference and CFX96 Real-Time PCR detection system (Bio-Rad).

### Cell transfection

Transformed BEAS-2B cells were transfected with LNA-anti-miR-301a or LNA-anti-control (Exiqon, Woburn, MA, USA) by using Lipofectamine RNAiMAX reagent. After 48 h, the cells were collected and performed the realtime PCR assayed to ensure silencing was achieved. The LNA-anti-control (anti-control-miR) was the miRCURY LNA miRNA inhibitor control Negative Control A (Exiqon, Woburn, MA, USA) and it has no more than 70% homology to any sequence in the mouse genome.

For knockdown of SMAD4, the shRNA1-Smad4: CTGCCAACTTTCCCAACAT; shRNA2-Smad4: GCCTCCCATTTCCAATCAT; shRNA3-Smad4: CGGTCTTTGTACAGAGTTA; and nonsense sequence to Smad4 (shRNA-control): GGTGTGCAGTTGGAATGTA were synthesized and ligase into psiF-copGFP vectors (System Biosciences, Mountain View, CA). 293 T cells were seeded in a 10 cm plate and transfected with 5 μg pRSV-rev, 5 μg pMDL-rre, 5 μg pMD2.G, and 10 μg shRNA plasmid construct. Then the cells were cultured at 37 °C for 48 h, and the virus was harvested by centrifuging the cells at 2000 g for 10 min followed by filtrating it through 0.45 μm filter. The BEAS-2B cells were transduced with 2 ml of the virus in the presence of 8 μg/mL of polybrene for 6 h, and then change the medium. After 48 h, the cells were collected and performed the western blot assay to ensure silencing was achieved.

### Cell proliferation and migration assay

Cell proliferation was determined by using Cell Counting Kit-8 (CCK-8) kit (Dojindo Molecular Technologies, Inc). Briefly, cells were seeded in 96-well plate and transfected with LNA-anti-miR-301a or shRNA-Smad4 with indicated concentration and time point. Cell viability was evaluated using WST-based CCK-8 assay (Dojindo) as described. For cell migration assay, cells were suspended in 1% FBS DMEM medium and incubated at 37 °C for 2 h and then placed in the upper chambers. The lower chambers were filled with DMEM with 5% FBS. After 24 h, the inserts were removed, and inner side was wiped with cotton swaps. The cells were fixed with 70% ethanol, stained with Giemsa and counted under light microscope.

### Colony formation assay

5 × 10^3^ cells were suspended in 0.3% agar in DMEM supplemented with 1% penicillin/streptomycin, 1% L-glutamine, and 10% FBS and overlaid on 0.5% agar in the same medium in six-well plates and maintained in an incubator for 4 weeks. Colonies were stained with 0.005% crystal violet, and greater than 0.1 mm in diameter were scored by microscopic examination and photographed with a dissection microscope. Arsenic-transformed cells from anchorage-independent colonies were selected and grown in DMEM and passage-matched cells with no arsenic treatment were used as the control.

### Cell apoptosis assay

BEAS-2B cells were transfected with the indicated miR-301a inhibitor or shRNA-Smad4 and negative control. Then the cells were stained with FITC Annexin-V apoptosis detection kit (Thermo Fisher) according to the manufacturer protocol and analyzed by flow cytometry (Accuri C6, BD).

### ELISA for IL-6 expression

IL-6 level in the supernatants from BEAS-2B cells were detected by using IL-6 ELISA Kit (Abcam, USA) according to the manufacturer’s instructions.

### Luciferase reporter assay

The 3′UTR of Smad4 was synthesized and sub-cloned into the pGL3-luciferase reporter vector. HEK-293 T cells were transfected with pGL3 vectors containing wild-type Renilla luciferase or firefly luciferase with Smad4 3′UTR and with 50 nM of mimic miR-301a or negative-control oligonucleotide (Exiqon). The cells were lysed, and luciferase activity was determined by using the dual luciferase reporter assay system (Promega, USA) 48 h after transfection.

### Xenograft model

For *in vivo* tumor formation, 5 × 10^5^ cells were injected subcutaneously into athymic nude mice. Tumor size was measure with calipers every 3 or 4 days, and the mice were euthanized at 18 days. Tumor volume was calculated by the formula of windth2 × length/2. All mice were kept under specific pathogen free (SPF) conditions and all mice experiments were approved by the ethical committee of South China Normal University and Guangzhou University of Chinese Medicine Institutional Animal Care and Use Committee and in accordance with the Guide for the Care and Use of Laboratory Animals (NIH).

### Western blot

For western blotting analyses, cells were lysed with RIPA buffer (Cell Signaling), supplemented with protease inhibitor (Calbiochem, Billerica, MA, USA). Protein concentrations were measured with the Pierce BCA Protein Assay Kit according to the manufacturer’s manual (Thermo Fisher Scientific, Waltham, MA, USA). Soluble proteins were subjected to SDS-polyacrylamide gel electrophoresis before being transferred to polyvinylidene difluoride (PVDF) membranes for blotting. The membrane was blocked with 5% nonfat dry milk for 1 hour and incubated with primary antibody at 4 °C overnight. The following antibodies were purchased from Cell Signaling Technology: phospho-Stat3 (pStat3, D3A7), Stat3(124H6), Smad4(D3R4N), IκBα(44D4), Runx3(D9K6L), Pten(D4.3), Bim(C34C5), P63(D9L7L), Pias3(D5F9) and Gadd45α (D17E8) and Nkrf (ab83977) was purchased from Abcam company. β-actin (1:5000) primary antibody was purchased from Sigma. After incubated with primary antibody, the membranes were incubated with either horseradish peroxidase conjugated anti-rabbit or anti-mouse secondary antibody. The immunoreactive proteins were visualized with SuperSignal West Dura Chemiluminescent (Thermo Fisher Scientific, USA). Quantitation of X-ray file from Fig. 4A was carried out by scanning densitometry using area integration. Other gel pictures from Figs 2D, 4D, 5A and 6F,G were scanned by automatic chemiluminescence imaging analysis system Tanon5200 and acquired using TanonPVCam software (Tanon).

### Data availability and Correlation analysis

The Cancer Genome Atlas (TCGA) miRNA-seq and RNA-seq data for lung adenocarcinoma and squamous cell carcinoma of cancer patients were downloaded from starbase.sysu.edu.cn^38^. For the correlation analyses between miR-301a and Smad4 gene expression, Pearson correlation coefficients were calculated.

### Statistical analyses

All statistical analysis was carried out using SPSS16.0 software (SPSS Inc. Chicago, IL, USA). An unpaired two-tailed Student’s t-test was performed for two-group comparisons and one-way ANOVA analysis was performed for multiple group comparisons. For the correlation analyses between miR-301a and Smad4 gene expression, Pearson correlation coefficients were calculated. The statistical significance was set at a P-value < 0.05 and values were presented as means ± SD.

## Data Availability

The Cancer Genome Atlas (TCGA) miRNA-seq and RNA-seq data for lung adenocarcinoma and squamous cell carcinoma of cancer patients were downloaded from starbase.sysu.edu.cn.
